# Decoding lymphangiogenesis in oral squamous cell carcinoma: Emphasis on clinical and histopathological determinants of regional metastasis

**DOI:** 10.1371/journal.pone.0311108

**Published:** 2024-11-14

**Authors:** Ankita Tandon, Kumari Sandhya, Narendra Nath Singh, Nikita Gulati, Amit Kumar, Kanika Sethi

**Affiliations:** 1 Department of Oral Pathology, Microbiology and Forensic Odontology, Dental Institute, RIMS, Ranchi, Jharkhand, India; 2 Department of Anatomy, RIMS, Ranchi, Jharkhand, India; 3 Department of Oral Pathology and Microbiology, ITS-CDSR, Muradnagar, Ghaziabad, UP, India; 4 Department of Lab Medicine, RIMS, Ranchi, Jharkhand, India; 5 Department of Oral Pathology and Microbiology, Inderprastha Dental College, Sahibabad, Ghaziabad, UP, India; University of California, Davis, UNITED STATES OF AMERICA

## Abstract

**Purpose:**

The growth and metastasis of solid epithelial tumors is lymphangiogenesis dependent. The most important lymphangiogenic inducers facilitating this progression is Vascular endothelial Growth Factor C (VEGF-C). The recent D2-40 (Podoplanin) antibody is specific for lymphatic epithelium and allows its objective assessment. Also, lymphovascular invasion (LVI) is a risk factor for lymph node metastases (LNM) and indicates a significant influx of tumor cells into the lymphatics, causing regional metastasis. Thus, the following study was conducted to assess and correlate VEGF-C and D2-40 immunoexpressions with clinical and histopathologic lymph node status in Oral Squamous Cell Carcinoma (OSCC) cases and also to estimate the impact of intratumoral (ILVD) and peritumoral lymphatic vessel density (PLVD) in such cases.

**Methodology:**

A total of 128 OSCC cases, divided as Group I: Cases with clinically and histopathologically negative lymph nodes (n = 64) and Group II: Cases with clinically negative but histopathologically positive lymph nodes (n = 64) were immunoscored for VEGF-C (Anti VEGF-C antibody) (PA5-29772, Invitrogen) and D2-40 (Anti D2-40 antibody) (IR072/8072, Dako) using standard protocols. The data was statistically evaluated using STATA 18.0 with p≤0.05 considered statistically significant throughout the study.

**Result:**

21.88% Group I cases and 40.62% Group II cases showed highest immuno-positivity for VEGF-C (p = 0.00). The mean D2-40 score for Intratumoral Lymphatic Vessel Density (ILVD) and Peritumoral Lymphatic Vessel Density (PLVD) was higher for group II cases (i.e., 41.5±13.73 and 35.95±8.27 respectively at 95% CI, p = 0.00) suggesting a direct correlation between Lymphatic Vessel Density (LVD) and LNM.

**Conclusion:**

Lymphangiogenesis is a true determinant of the biologic potential of OSCC and obtaining an objective data in terms of LVD through D2-40 could be impactful in OSSC diagnosis and guiding treatment decisions by clinicians.

## Introduction

Global Cancer Observatory (GCO) data indicates that in 2020, there were 377,713 yearly cases of OSCC worldwide [1]. With 248,360 instances, Asia had the highest number, followed by North America (27,469) and Europe (65,279). Based on their anatomical location, the many types of cancers that affect the head and neck are categorized by the World Health Organization’s (WHO) International Classification of Diseases (ICD-10) system. Roughly 90% of Head and Neck Cancers (HNCs) are squamous cell carcinomas (SCCs), which arise from the epithelial lining of the larynx, throat, and mouth [1, 2].

It was projected that in 2020, oral cancer will rank as the sixteenth most common cause of cancer-related fatalities worldwide, with a significant proportion of deaths from the illness occurring in men in South and Southeast Asia and the Western Pacific. Numerous researchers have examined and proposed different prognostic implications for histopathologic and clinicopathologic indicators of OSCC [3] considering that the epidemiologic data regarding the disease burden is frightening enough. Since OSCC is an epithelial malignancy, despite the fact that several pathogenetic models have been postulated, the actual biologic potential of the disease in terms of its progression can only be determined by the tumor cells spreading through lymphatics. Only a fraction of existing literature focuses upon this true determinant in terms of Lymphatic Vessel Density (LVD), Lymphovascular Invasion (LVI), ILVD, PLVD etc. in primary tumor and is not sufficient to claim the hypothesis of how existing (previous lymphatic channels) or neo-lymphangiognesis (formation of new lymphatic channels) contribute to the process.

LVI, the most researched parameter, refers to tumor cells that invade a portion of a vascular or lymphatic channel that is bordered by endothelium but does not have underlying muscle walls. Tumor cells enter lymphovascular spaces through the endothelium cell layer, which is a critical stage in the spread of malignancies. Nevertheless, statistical evidence indicates that LVI must be evaluated since its presence in randomly selected tissue sections indicates that a high number of tumor cells are entering the vascular compartment, raising the possibility of metastasis [4]. As per the most widely accepted theory, the growth of the tumor triggers the production of lymphangiogenic growth factors, mainly from the VEGF family, which in turn causes the formation of new vessels and the incorporation of pre-existing lymphatic vessels within the tumor cell sheets. Next, cancer cells move into the lymphatic vessels by following the route of least resistance after separating from the growing tumor mass. After attaching to lymphatic endothelium, cancer cells cross the endothelial barrier and enter the lymphatic lumen [5].

LVI is also an early indicator of metastasis and also a risk factor for lymph node metastases [6]. Its existence suggests a substantial inflow of tumor cells into the vascular compartment, one of the first steps toward the potential metastasis’s development [7]. Although LVI has been included to the eighth AJCC staging system as an additional prognostic factor, research is still being done to see whether it can be used to stratify OSCC patient risk for survival or recurrence [7].

Despite the importance of lymphogenic metastases in OSCC, numerous concerns remain about the molecular processes underlying the lymphatic-tumor relationship. First, how much neo-lymphangiogenesis (formation of new lymphatic channels) in OSCC is induced and whether most of these new lymphatic vessels are located in intratumoral or peritumoral areas are the major areas of concern. The principal pathway for the spread of cancer cells, the intratumoral or peritumoral lymphatic system, is another aspect to take into account. While peritumoral lymphatic channels seem to be the main pathway for cancer cell dissemination, recent evidence suggests that intratumoral lymphatics should be taken into account as an additional route for LNM [8].

Studies show lymphangiogenesis is crucial for solid epithelial cancers, with VEGF-C being a key inducer. Also, D2-40, can detect lymphatic epithelium specifically. The findings of this study may provide an important addition to the currently used prognostic parameters that are assessed in a primary tumor. A broader view towards lymphangiogenesis needs to be undertaken in patients undergoing neck dissections. Despite of significant advancements in this regard, surgical treatment (treatment of choice) incorporating neck dissection is associated with significant amount of mortality and morbidity. Furthermore, the research gap based on assessment of true lymphangiogenesis in OSCC cases needs closure. Therefore, efforts to establish an autocrine loop involving lymphatic vascular density may be worked upon routinely, which would help to raise our understanding of the lymphangiogenic interactions in OSCC cases and their impact on loco-regional metastasis thereby identifying their potential utility as prognostic or interventional targets. This approach may help in predicting tumor negative lymph nodes through lymphangiogenic markers and hence sparing a significant number of patients from the morbidity associated with extensive surgical interventions.

## Methodology

### Patients and tissue samples

This retrospective cohort study was conducted on archival tissue samples which were submitted for histopathological evaluation in the Department of Oral Pathology, Microbiology and Forensic Odontology after gaining consent from the Institutional Ethics Committee, RIMS, Ranchi (vide letter no. 20, dated 03/02/2022). The archival samples and their corresponding data were accessed for research purposes between 03/03/2022 to 15/12/2023. All the corresponding data from patient’s records were fully anonymized during the entire study (all data uploaded as S1 Data). All procedures performed in the study were in accordance with the 1964 Helsinki declaration and its later amendments or comparable ethical standards [9]. Study samples consisted of 128 excisional biopsy cases in total, which included Group I: OSCC cases with clinically and histopathologically negative lymph node cohort (n = 64) and Group II: OSCC cases with clinically negative but histopathologically positive lymph node cohort (n = 64). All cases were clinically staged using AJCC level-based nodal classification which defines TNM (Tumor Node Metastasis) as Stages I, II, III, and IV and histopathologically graded using WHO 2005 criteria as well differentiated, moderately differentiated, poorly differentiated, and anaplastic cases. Every case’s clinical information, including TNM staging, was gathered from patients’ records. Patients who had not had any prior treatment were included in the study, as were patients who underwent radiation or surgery (radical or selective neck dissection) as the first line of treatment. Patients with significant necrosis and/or superadded infection, prior chemotherapy, patients who declined surgical treatment, patients for whom treatment other than surgery was recommended, and additional concurrent primary tumors were eliminated.

### Immunohistochemistry with VEGF-C and D2-40 Anti D2-40

Three-micrometer-thick sections from archival formalin-fixed paraffin-embedded tissues were placed on poly-l-lysine-coated slides for immunohistochemistry. VEGF-C (Anti VEGF-C (PA5-29772, Invitrogen) and D2-40 Anti D2-40 (IR072/8072, Dako) immuno expressions were analysed by immunohistochemical examination with antibodies. For VEGF-C immunostaining, prostate served as the positive control whereas for D2-40 immunostaining, Breast cancer served as positive control. The negative control was performed by omitting the primary antibodies for both the immunostaining procedures. We used mouse monoclonal anti-VEGF-C antibody diluted at 1:50. Tris-EDTA buffer antigen retrieval protocol was used to unmask epitopes in formalin-fixed and paraffin embedded tissue sections. pH Tris EDTA epitope retrieval was 6, while primary antibody was incubated at room temperature overnight. For D2-40 monoclonal antibody endogenous peroxidase was blocked by immersion in methanol containing 3% hydrogen peroxide for 5 min. After a 5-min wash with PBS, the nonspecific binding was blocked in PBS containing 1% bovine serum albumin at room temperature for 30 min. The blocked sections were incubated at 4°C overnight with 50-fold diluted monoclonal antibody D2-40 in PBS, and D2-40 binding was visualised using an avidin-biotin complex immunoperoxidase procedure.

### Assessment of immunoscoring of cell positivity and intensity

Based on whether or not they had LVI, the patients were split into two groups (V−/V +). A microvessel was defined as an individual or a group of positive podoplanin-expressing endothelial cells that surrounded a visible lumen and could be clearly distinguished from other connective tissue elements and neighboring microvessels. Peritumoral lymphatic vessels were identified as those in the periphery within 2 mm of tumors next to the invasion front, while intratumoral lymphatic vessels were characterized as those within the tumor cell islets. In short, a 40× field was used to mainly display the three most vascularized locations that podoplanin examined-also referred to as hotspots. Next, vessels in each of these regions were found using a 200×field of view [10].

VEGF-C expression was evaluated using a light microscope by two pathologists, blinded to the information of clinical outcomes. Scoring was done based on the proportion and intensity of staining in tumor cell cytoplasm. The intensity of staining was evaluated as follows: 0, no staining; 1, weak; 2, moderate; and 3, strong. The proportion of positive tumor cells was scored as 0 (No positive cells); 1 (1–10% positive cells); 2 (11–49% positive cells); 3 (> 50% positive cells) [11].

### Statistical analysis

Statistical analysis was performed using STATA 18.0 SE-Standard Edition (Stata Corp LLC, Texas-USA 800-STATA-PC 979-696-4600, Serial Number 401806362156). The descriptive statistics was reported in the form of frequency, percentages, mean and standard deviation. Normal distribution of the data having ratio scale was evaluated by histogram, skewness, kurtosis, Kolmogorov ‐ Smirnov Test and Shapiro Wilk Test. The morphological score was compared between Group I (clinically and histopathological negative LN) and Group II (clinically negative and histopathological positive LN) by Mann Whitney U Test. Pearson’s and Spearman’s correlation was used to evaluate relationship between VEGF-C and D2-40 immunoexpression with the morphological scores in both groups depending on the distribution of the data. p ≤ 0.05 was considered statistically significant throughout the study.

## Results

### Demographic data

The overall mean age of the study cases was 46.13 years. The cases included in the study had a higher male representation, i.e., 87.5%. Also, 78.12% cases were males in Group I category and 76.56% cases were males in Group II category. Majority of cases were cited on buccal mucosa (27.34%) followed by alveolar mucosa (18.75%), gingivobuccal sulcus (17.19%), buccal vestibule (13.28%), lateral border of tongue (11.72%), labial vestibule (7.03%), labial mucosa (3.91%) and lingual vestibule (0.78%) (p = 0.010) respectively.

### Description of cases

**Fig 1:**
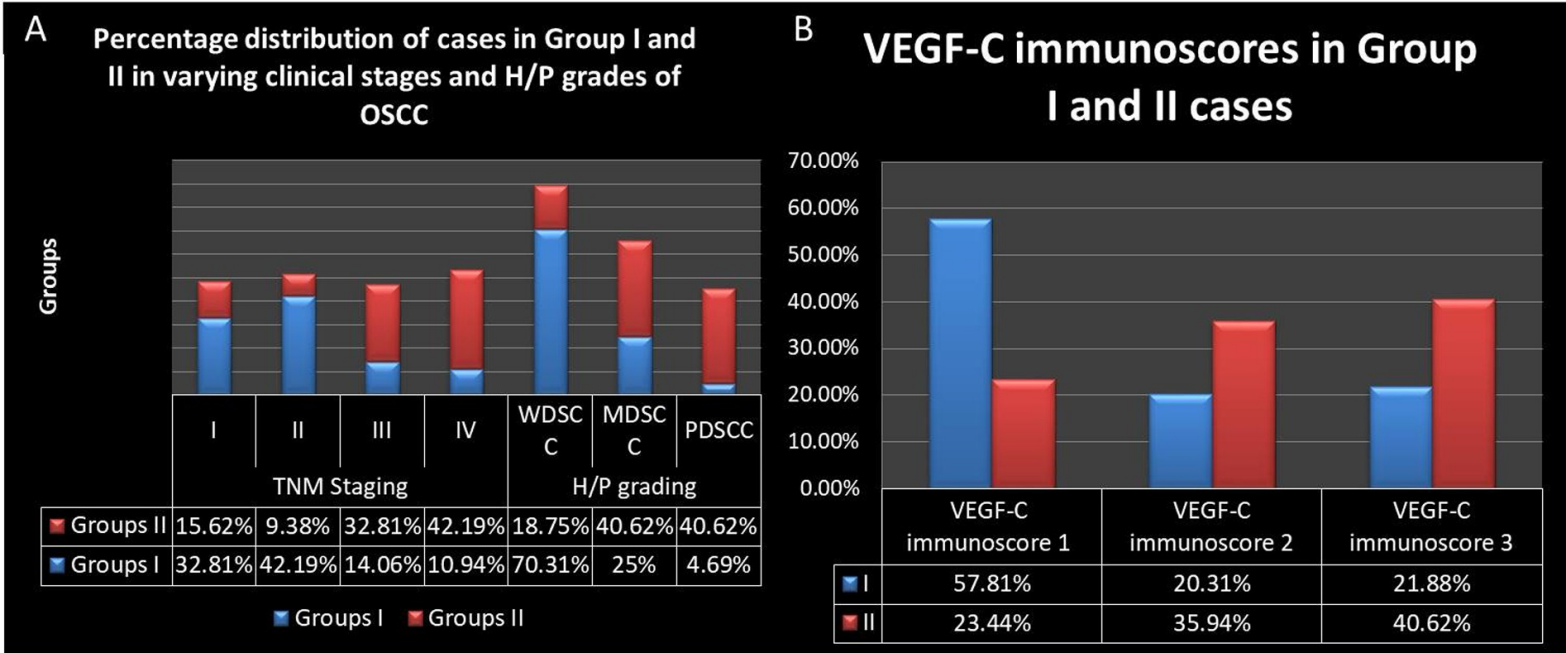
(A) Percentage distribution of cases in both groups corresponding to all clinical and histopathological categories of OSCC. (B) VEGF-C immunoscores in both groups.

In our study, 42.19% cases of Group I were Stage II of the disease in comparison to majority of cases (42.19%) of Group II which were Stage IV. Majority of Group I cohort were histopathologically confirmed as well differentiated SCC (70.31%) and belonged to Stage II OSCC (42.19%) whereas Group II showed equal preponderance of moderately and poorly differentiated SCC (40.62%) and majority of such cases belonged to stage III OSCC (32.81%) **(Fig 1A).**

### Expression of VEGF-C in OSCC

**Fig 2:**
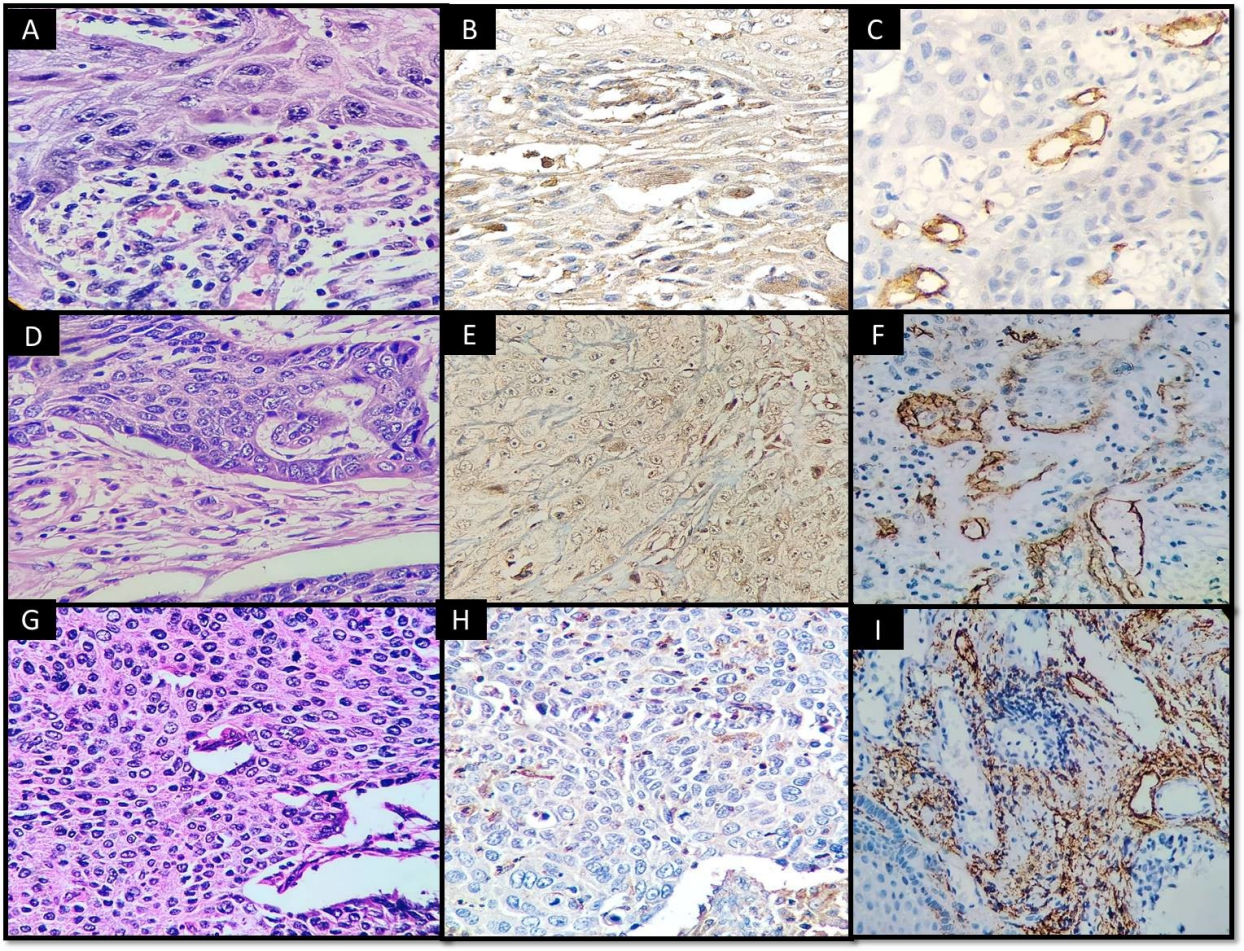
VEGF-C and D2-40 immunoexpressions in well differentiated OSCC (A, B, C); Moderately differentiated OSCC (D, E, F) and Poorly differentiated OSCC (G, H, I).

100% cases in our study showed VEGF-C immuno-positivity. 21.88% Group I cases and 40.62% Group II cases showed highest immuno-positivity for VEGF-C (p≤0.05) **(Fig 2)**. Majority of the cases of Group I (57.81%), showed immunoscore 1 of VEGF-C whereas majority of the cases of Group 2 (40.62%) showed immunoscore 3 **(Fig 1B).**

**Fig 3:**
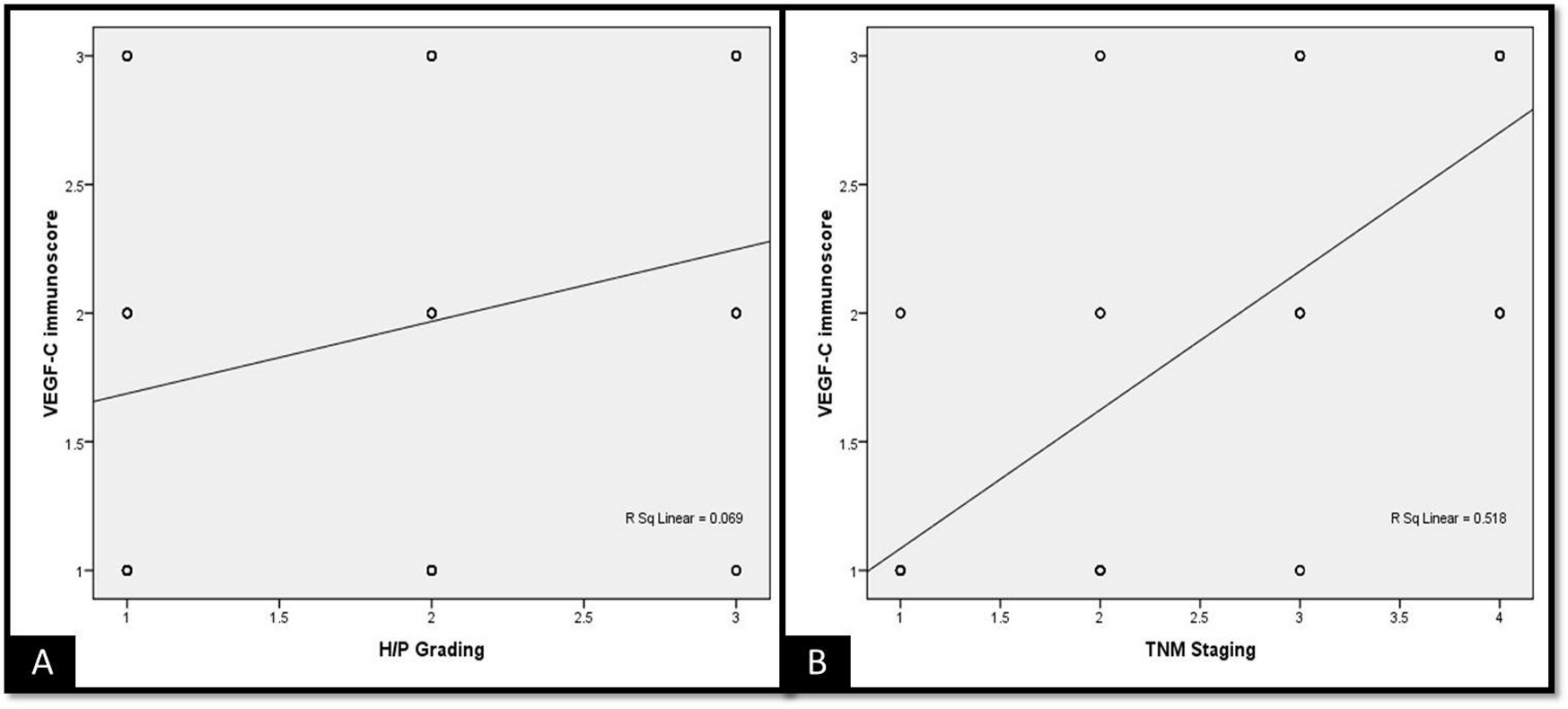
Scatter plots depicting correlation between (A) VEGF-C and histopathological grading of OSCC cases and (B) VEGF-C and TNM (clinical) staging of OSCC cases.

The immunoexpression of VEGF-C increased with increasing grades of histopathological scores as well as TNM staging. Although the immunoscores of VEGF-C were not significant among the study groups (**Fig 3).**

### Expression of D2-40 in OSCC

**Fig 4:**
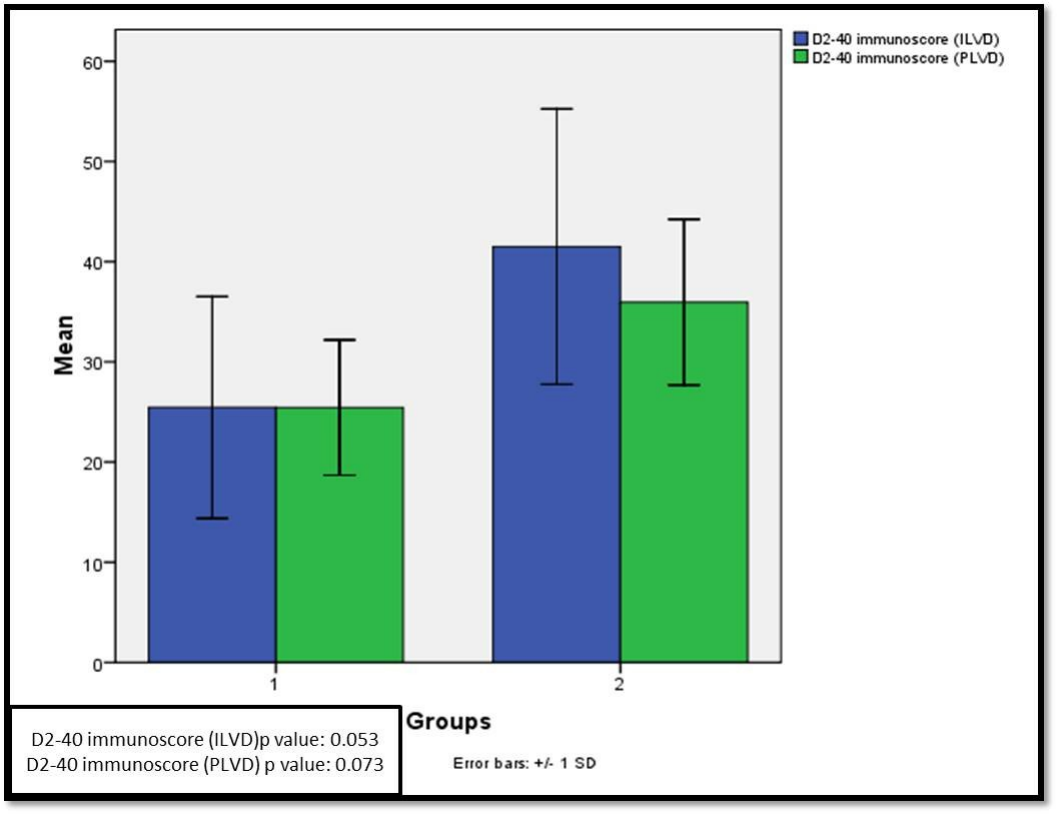
Mean D2-40 immunoscores (ILVD and PLVD) in both groups.

100% cases in our study showed D2-40 immuno-positivity. ILVD for group I was 25.45313 ±1.384246 whereas; for group 2 was 41.5 ± 1.71723. On the other hand, PLVD for group I was 25.43±6.74 whereas for group 2 were 35.95±8.27 **(Fig 4).**

### ILVD and PLVD scores

**Fig 5:**
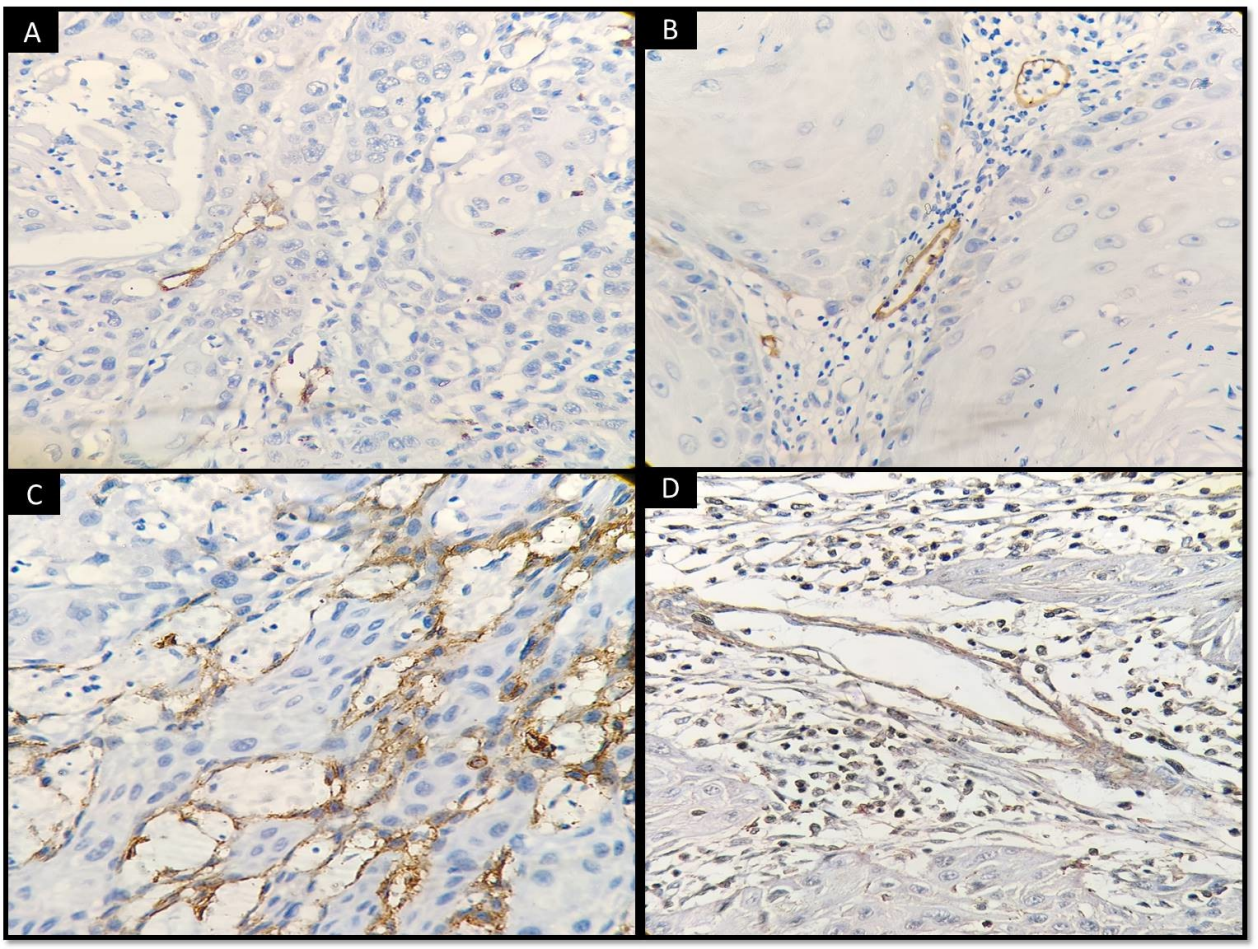
Intratumoral (A, B) and peritumoral (C, D) lymphatic vessels in OSCC cases.

As regards to objective assessment of lymphangiogenic potential amongst all cases the mean D2-40 score for ILVD and PLVD was higher for group II cases (i.e., 41.5±13.73 and 35.95±8.27 respectively at 95% CI, p = 0.00) suggesting a direct correlation between LVD and lymph node metastasis **(Fig 5).**

**Fig 6:**
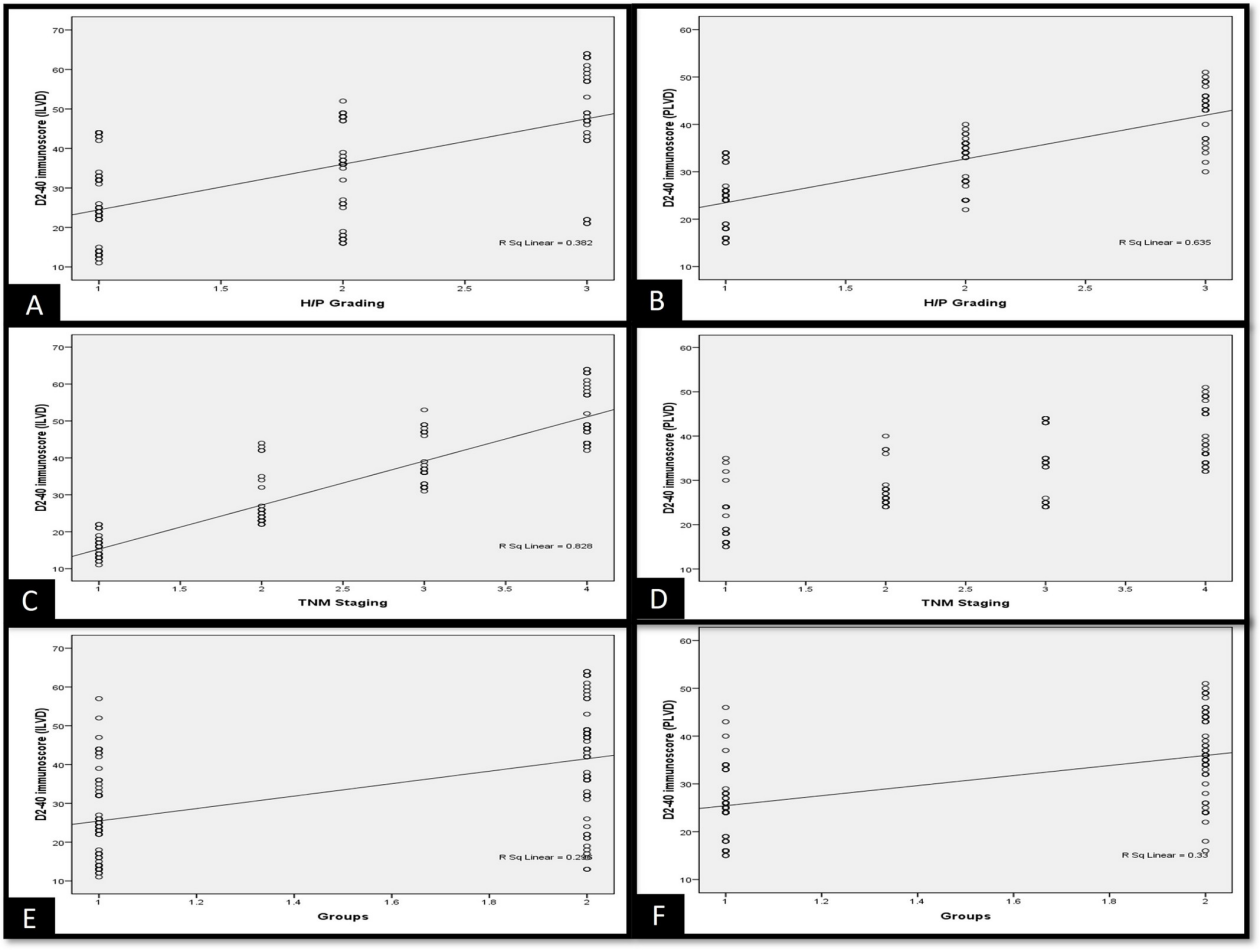
Scatter plots depicting correlation between D2-40 immunoscores (ILVD-A, C, E) (PLVD-B, D, F) with histopathological assessment, clinical assessment, and between groups of OSCC cases.

There is a positive correlation between D2-40 intratumoral and peritumoral lymphatic vessel density with histopathological as well as clinical grades of OSCC (p≤0.05) **(Fig 6).**

## Discussion

Tumor invasion of lymphatic vessels has long been thought to be a major pathogenic factor in OSCC metastasis. However, its impact on recurrence and tumor locoregional control remains uncertain. Jakobsson et al. [12] included an evaluation of the degree and presence of LVI in the multifactorial grading system. Its existence suggests a substantial inflow of tumor cells into the vascular compartment, one of the first steps toward the potential metastasis’s development. The principal pathway for which involves enhancement of ILVD and PLVD which is a significant component contributing to the way that oral cancer cells enter lymphatics [13].

The most commonly recognised explanation states that a tumour creates lymphangiogenic growth factors, mostly members of the VEGF family, as it expands. Following that, these variables cause lymphatic vessels that are already existing within tumour cell sheets to be included as well as new vessels to develop. By taking the path of least resistance, cancer cells migrate away from the expanding tumour mass and towards the lymphatic capillaries [14].

How lymphovascular invasion influences OSCC patients’ response to treatment is yet unclear. Concerns over the influence of LVI on treatment failure were raised by Fagan et al.’s [15] observation that vascular and lymphatic invasion were not significantly associated with local recurrence in patients with head and neck SCC. This conclusion was supported by the results of Tai et al. [16] regarding locoregional control of early-stage tongue OSCC and Chen et al. [17] regarding disease control of early-stage OSCC. However, some researchers have linked LVI to locoregional recurrence or distant metastasis. Their study, which is the first to examine vascular and lymphatic invasion independently, found that neither invasion type has an impact on locoregional recurrence after treatment or distant metastasis [6].

Whereas the majority of intratumoral lymphatic vessels in our study were small and collapsed, LVs around the invasive edge were often bigger and dilated. Our research supported the findings of Franchi et al. [18] who discovered a statistically significant increase in peritumoral lymphatics in cases of HNSCC, including metastases to lymph nodes. Consequently, they proposed that peritumoral lymphangiogenesis in patients with HNSCC may indicate the possibility of lymph node metastases [19].

**Fig 7:**
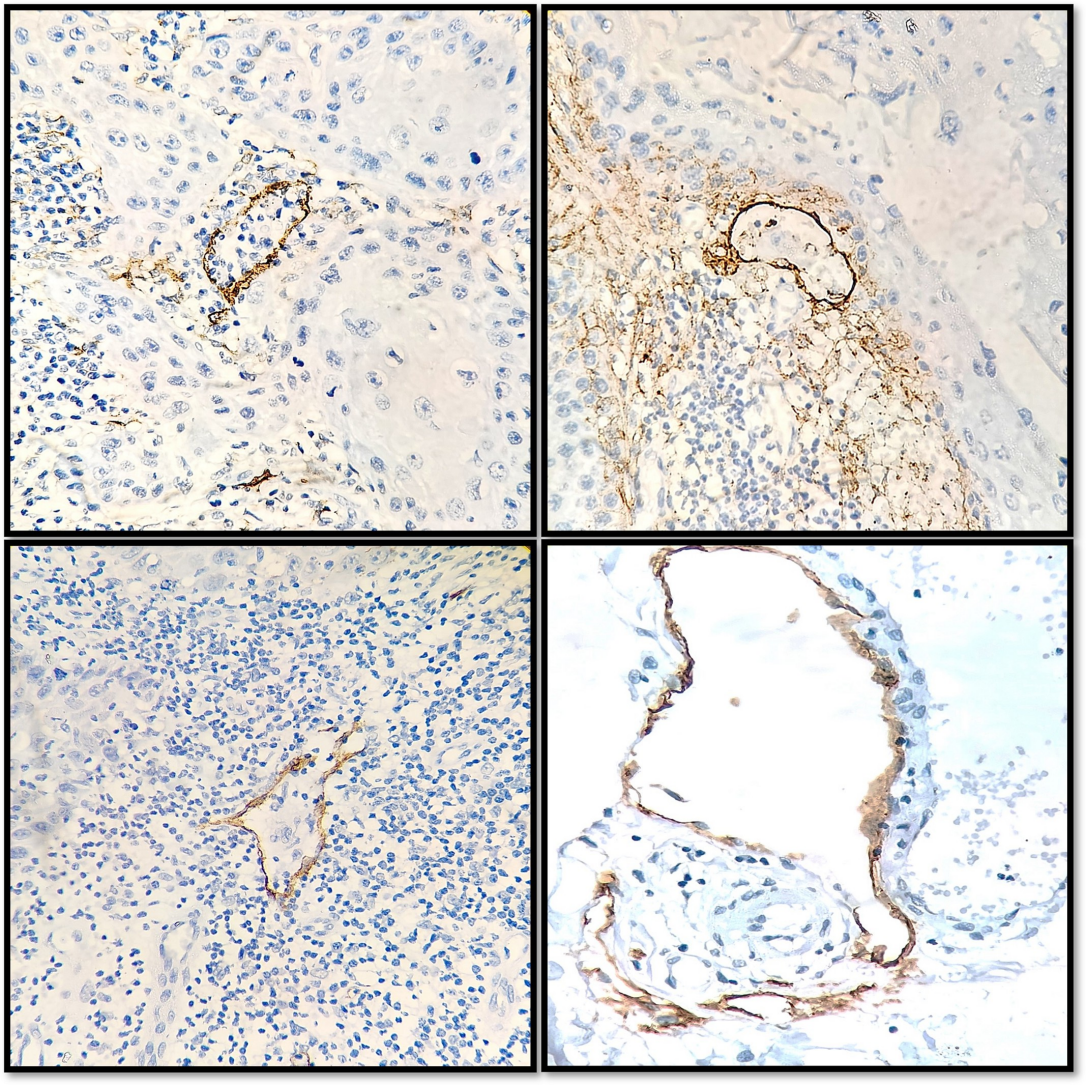
A few representations of lymphovascular invasion.

Our observations suggest that poorly differentiated OSCC may benefit from podoplanin expression along the invasive front due to their apparent tendency for local development and lymphovascular invasion **(Fig 7).** Thus, there is a relationship between a greater likelihood of lymph node metastases and elevated podoplanin levels. In lesions showing localized expression of podoplanin, the outside layer of the tumor cells showed positive staining, but the inner cell nests showed no staining at all. Well-differentiated OSCC showed a more pronounced diffuse staining pattern than poorly differentiated OSCC, which was more common, especially in the invasive front areas. These results may be explained by the theory of tumor initiating cells, which was supported by the study of a few other authors. VEGF-C epithelial expression was greater in positive node OSCC cases compared to node-free cases [19].

Research by Sugiura et al. [20] and Yanase et al. [21] revealed an increased VEGF-C immunoexpression in tumors with invasive front regions, especially in the positive node OSCC. These results raised the possibility that VEGF-C promotes the invasiveness of cancer cells. According to Sugiura et al. [20] VEGF-C’s capacity to stimulate urokinase synthesis by cancer cells expedites plasmin-mediated matrix breakdown, hence facilitating the cancer cells’ proliferation and invasion of adjacent tissue, which explains the rise in VEGF-C expression at the invasive front. Through a paracrine mechanism, VEGF-C promotes endothelial cell proliferation and sprouting. It was demonstrated that VEGF-C binds to VEGFR-2 and VEGFR-3, which are found on lymphatic and blood endothelial cells, respectively [21].

Moreover, a variety of chemokines that regulate leukocyte aggregation in the tumor-associated stroma are released by endothelial cells as a result of VEGF-C. Reactive oxygen species, matrix metalloproteinases (MMPs), and chemokines released by VEGF-C control the growth, invasion, angiogenesis, and proliferation of cells. Consequently, VEGF-C plays a critical role in the remodeling of the extracellular matrix through the release of urokinase by cancer cells as well as the release of MMPs by leukocytes that VEGF-C attracts. Moreover, it has been established that VEGF-C acts as a pro-lymphangiogenic agent, causing the lymphatic endothelium to express VEGFR-3. This is supported by studies conducted in a mouse model, which demonstrated that blocking VEGFR-3 signaling using fusion proteins that act as VEGF-C’s traps reduced tumor lymphangiogenesis and lymphatic dispersion [12, 13].

The authors have made an attempt to determine the purpose and impact of lymphangiogenesis in the present analysis. However, there are a few strengths and limitations of the study which may be listed as:

### Strengths

1. The study addresses the research gap and scarcity of information about the mechanisms of lymphangiogenesis and its significance in loco regional spread through a two-step mechanism to identify true lymphangiogenic potential of the primary tumor.
2. D2-40 clearly discriminates lymphatic vessels from blood vessels, thereby removing the possibility of pseudo identification.
3. By measuring lymphatic vessel density, the study offers an objective evaluation of lymphatic potential of the tumor.
4. The study’s information on cancer-mediated lymphangiogenesis will aid the researchers to refocus their attention and find more effective treatment approaches for OSCC by identifying newer therapeutic targets and reducing the morbidity associated with conventional treatment approaches.
5. The results of this study might significantly improve the prognostic factors currently employed to evaluate primary epithelial malignancies eg. OSCC.

### Limitations

1. The study does not correlate the primary tumor microenvironment in relation to lymphangiogenesis. The author’s plan to study the same in further extensions of this study.
2. The study also did not pay emphasis on lymph node architectural changes in detail as they may hold prognostically relevant information. The authors have planned to evaluate the same in future and formulate a clinic-histopathological model of OSCC based on detailed findings.

## Conclusion

Given that lymphangiogenesis is a dependable predictor of the biologic potential of OSCC, it might help surgeons select the most appropriate treatment approach based on the unique risk profile of each patient (Group I vs. Group II). Decoding the impact of intratumoral and peritumoral lymphatic vascular density on OSCC is critical to implementing meaningful modifications to treatment selection and procedure protocols for OSCC and lowering the morbidity burden associated with needless surgical interventions especially in cases with N_0_ neck.

## Supporting information

S1 Data (XLSX)

## References

[1] Sung H, Ferlay J, Siegel RL, Laversanne M, Soerjomataram I, Jemal A, et al. Global Cancer Statistics 2020: GLOBOCAN Estimates of Incidence and Mortality Worldwide for 36 Cancers in 185 Countries. CA Cancer J Clin. 2021; 71: 209–249. doi: 10.3322/caac.21660 33538338

[2] Johnson DE, Burtness B, Leemans CR, Lui VWY, Bauman JE, Grandis JR. Head and neck squamous cell carcinoma. Nat Rev Dis Primers. 2020;6: 92. doi: 10.1038/s41572-020-00224-3 33243986 PMC7944998

[3] Bouvard V, Nethan ST, Singh D, Warnakulasuriya S, Mehrotra R, Chaturvedi AK, et al. IARC Perspective on Oral Cancer Prevention. N Engl J Med. 2022;387: 1999–2005. doi: 10.1056/NEJMsr2210097 36378601

[4] Khan SJ, Gawande M, Hande AH, Patil SK, Sonone AM. Correlation of Pattern of Invasion, Stromal Inflammation and Lymphovascular Invasion with Histopathological Grading in Oral Squamous Cell Carcinoma: A Retrospective Study. Cureus. 2024;16: e52233. doi: 10.7759/cureus.52233 38352087 PMC10861803

[5] Mascitti M, Togni L, Caponio VCA, Zhurakivska K, Bizzoca ME, Contaldo M, et al. Lymphovascular invasion as a prognostic tool for oral squamous cell carcinoma: a comprehensive review. Int J Oral Maxillofac Surg. 2022;51: 1–9. doi: 10.1016/j.ijom.2021.03.007 33814227

[6] Choi S, Song JH, Lee S, Cho M, Kim YM, Kim HI, et al. Lymphovascular Invasion: Traditional but Vital and Sensible Prognostic Factor in Early Gastric Cancer. Ann Surg Oncol. 2021;28: 8928–8935. doi: 10.1245/s10434-021-10224-6 34075484

[7] Adel M, Kao HK, Hsu CL, Huang JJ, Lee LY, Huang Y, et al. Evaluation of Lymphatic and Vascular Invasion in Relation to Clinicopathological Factors and Treatment Outcome in Oral Cavity Squamous Cell Carcinoma. Medicine (Baltimore). 2015;94: e1510. doi: 10.1097/MD.0000000000001510 26512553 PMC4985367

[8] Abdul-Aziz MA, Amin AK, El-Rouby DH, Shaker OG. Lymphangiogenesis in Oral Squamous Cell Carcinoma: Correlation with VEGF-C Expression and Lymph Node Metastasis. Int J Dent. 2017;2017: 7285656. doi: 10.1155/2017/7285656 28680444 PMC5478861

[9] Carlson RV, Boyd KM, Webb DJ. The revision of the Declaration of Helsinki: past, present and future. Br J Clin Pharmacol. 2004;57:695–713. doi: 10.1111/j.1365-2125.2004.02103.x 15151515 PMC1884510

[10] Royston DJ, Clasper S, Jackson DG. Immunohistochemical Methods for Measuring Tissue Lymphangiogenesis. Methods Mol Biol. 2016;1430: 35–48. doi: 10.1007/978-1-4939-3628-1_2 27172944

[11] Sahaf R, Rehman A, Hussain S, Rasool G, Anjum S, Nagi AH, et al. Expression of Vascular Endothelial Growth Factor-C in oral squamous cell carcinoma: An immunohistochemical study. Biomed J Sci & Tech Res. 2018;2: 2079–2084. doi: 10.26717/BJSTR.2018.02.000626

[12] Jakobsson PA, Eneroth CM, Killander D, Moberger G, Mårtensson B. Histologic classification and grading of malignancy in carcinoma of the larynx. Acta Radiol Ther Phys Biol. 1973;12: 1–8. doi: 10.3109/02841867309131085 4725642

[13] Huang S, Zhu Y, Cai H, Zhang Y, Hou J. Impact of lymphovascular invasion in oral squamous cell carcinoma: A meta-analysis. Oral Surg Oral Med Oral Pathol Oral Radiol. 2021;131: 319–328.e1. doi: 10.1016/j.oooo.2020.10.026 33309267

[14] Cote B, Rao D, Alany RG, Kwon GS, Alani AWG. Lymphatic changes in cancer and drug delivery to the lymphatics in solid tumors. Adv Drug Deliv Rev. 2019;144: 16–34. doi: 10.1016/j.addr.2019.08.009 31461662

[15] Fagan JJ, Collins B, Barnes L, D’Amico F, Myers EN, Johnson JT. Perineural invasion in squamous cell carcinoma of the head and neck. Arch Otolaryngol Head Neck Surg. 1998;124: 637–40. doi: 10.1001/archotol.124.6.637 9639472

[16] Tai SK, Li WY, Chu PY, Chang SY, Tsai TL, Wang YF, et al. Risks and clinical implications of perineural invasion in T1-2 oral tongue squamous cell carcinoma. Head Neck. 201234: 994–1001. doi: 10.1002/hed.21846 21953773

[17] Chen JC, Chang YW, Hong CC, Yu YH, Su JL. The role of the VEGF-C/VEGFRs axis in tumor progression and therapy. Int J Mol Sci. 2012;14: 88–107. doi: 10.3390/ijms14010088 23344023 PMC3565253

[18] Franchi A, Gallo O, Massi D, Baroni G, Santucci M. Tumor lymphangiogenesis in head and neck squamous cell carcinoma: a morphometric study with clinical correlations. Cancer. 2004;101: 973–8. doi: 10.1002/cncr.20454 15329906

[19] Sharma G, Kumar R, Singh HP, Gupta M, Gupta M. Expression of podoplanin in tumor cells and lymphatic vessels in both tumoral and peritumoral areas and correlation with metastatic potential of oral squamous cell carcinoma. J Oral Maxillofac Pathol. 2021;25: 131–135. doi: 10.4103/jomfp.jomfp_481_20 34349423 PMC8272477

[20] Sugiura T, Inoue Y, Matsuki R, Ishii K, Takahashi M, Abe M, et al. VEGF-C and VEGF-D expression is correlated with lymphatic vessel density and lymph node metastasis in oral squamous cell carcinoma: Implications for use as a prognostic marker. Int J Oncol. 2009;34: 673–80. doi: 10.3892/ijo_00000193 19212672

[21] Yanase M, Kato K, Yoshizawa K, Noguchi N, Kitahara H, Nakamura H. Prognostic value of vascular endothelial growth factors A and C in oral squamous cell carcinoma. J Oral Pathol Med. 2014;43: 514–20. doi: 10.1111/jop.12167 24762199

